# Successful Management of Calciphylaxis with Sodium Thiosulfate in End-Stage Renal Disease: A Case Report

**DOI:** 10.3390/healthcare13030282

**Published:** 2025-01-31

**Authors:** Mohamed A. Albekery, Munirah K. Alkulaib, Ahmed A. Alanazi, Lulwah T. Alturki, Muthana A. Al Sahlawi, Ramy I. Abulikailik, Elbadri I. Abdelgadir

**Affiliations:** 1 Department of Pharmacy Practice, College of Clinical Pharmacy, King Faisal University, P.O. Box 400, Hofuf 31982, Saudi Arabia; munirahalkulaib@gmail.com; 2 Department of Pharmacy Practice, College of Clinical Pharmacy, Imam Abdulrahman Bin Faisal University, Dammam 34221, Saudi Arabia; aasalanazi@iau.edu.sa; 3 Department of Medicine, King Abdulaziz Hospital, Ministry of National Guard Health Affairs, Hofuf 31982, Saudi Arabia; alturkilu@ngha.med.sa (L.T.A.); ramys44@hotmail.com (R.I.A.); abdelgaderb@ngha.med.sa (E.I.A.); 4 Department of Internal Medicine, College of Medicine, King Faisal University, Hofuf 31982, Saudi Arabia; mualsahlawi@kfu.edu.sa

**Keywords:** calciphylaxis, sodium thiosulfate, end-stage renal disease, case report

## Abstract

Introduction: Calciphylaxis, also known as calcific uremic arteriolopathy (CUA), is a rare and potentially fatal condition primarily affecting patients with end-stage renal disease (ESRD) on hemodialysis (HD). It is characterized by calcification in small blood vessels, leading to painful skin ulcers and high mortality rates. Case Description: This is a case of a 42-year-old female with ESRD on HD who developed calciphylaxis, presenting with non-healing ulcers on her thighs. Discussion: Despite initial treatments, including wound care and pain management, her condition did not improve. A skin biopsy was inconclusive, highlighting the diagnostic challenges associated with calciphylaxis. Based on clinical judgment, warfarin and calcium-based therapies were discontinued, and the patient’s HD regimen was adjusted. Due to the persistence of symptoms, sodium thiosulfate (STS) therapy was initiated, leading to significant improvement in her ulcers after six months of treatment. Conclusions: This case highlights the importance of clinical judgment in the diagnosis and management of calciphylaxis, particularly when histopathological diagnostic methods yield inconclusive results. Clinical criteria, alongside a thorough assessment of the patient’s history and presentation, are vital for achieving a timely diagnosis in such challenging cases. The successful use of sodium thiosulfate in this patient adds to the growing body of evidence supporting its use as a potential treatment for calciphylaxis, especially in cases that do not respond to conventional therapy.

## 1. Introduction

Calciphylaxis, also known as calcific uremic arteriolopathy (CUA), is a rare and life-threatening complication with an unclear mechanism. It is characterized by the calcific occlusion of the medial layer of arterioles and small arteries in the subcutaneous adipose tissue and dermis [1,2]. Commonly affected areas include the abdomen, thighs, buttocks, and digits [1]. Patients typically present with lesions, tissue ischemia, necrosis, intense pain, or sepsis, which can lead to death. The prognosis for calciphylaxis is poor, with a one-year mortality rate estimated to be between 45% and 80% [3,4]. In 2014, Fresenius Medical Care North America (FMCNA) reported an incidence rate of 3.49 cases of calciphylaxis per 1000 patient–years in their dialysis units [5,6]. The exact prevalence is unknown due to its rarity, but small studies have reported a prevalence of up to 4% in patients undergoing hemodialysis [7]. This serious complication primarily affects patients with end-stage renal disease (ESRD) who are on hemodialysis (HD) or peritoneal dialysis (PD), particularly those who are female, diabetic, obese, have elevated serum phosphorus and calcium levels, decreased albumin levels, or are on therapies such as warfarin, calcium salts, or vitamin D therapies [4,8]. We reported a case of calciphylaxis in a young female patient with ESRD on HD, who was successfully managed with sodium thiosulfate (STS), highlighting the diagnostic challenges and therapeutic options available for this condition.

## 2. Case Description

**Figure 1:**
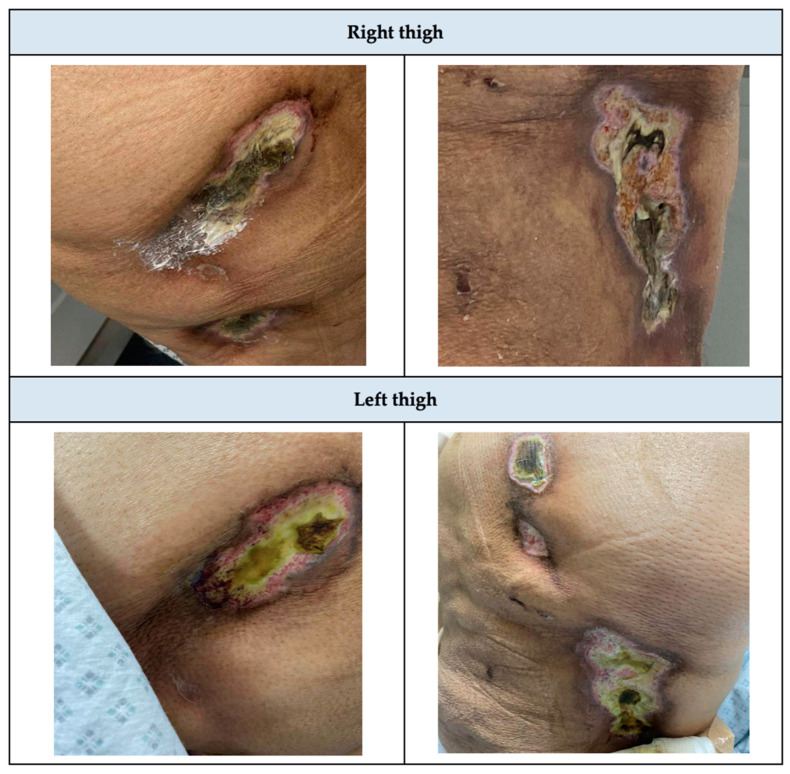
Initial presentation of Lateral thighs before calciphylaxis treatment.

A 42-year-old female with a known case of type-2 diabetes mellitus (T2DM), hypertension (HTN), bronchial asthma, biopsy-proven diabetic kidney disease, ESRD on HD for 3 years, a provoked deep venous thrombosis (DVT) that occurred in the right upper extremity 2 years ago, and obesity. The patient’s home medications were etelcalcetide 5 mg intravenous (IV) pushed three times a week (3/wk), alfacalcidol 2 mcg IV pushed 3/wk, darbepoetin alfa 40 mcg IV pushed 1/wk, sevelamer 800 mg orally (PO) twice a day (BID), warfarin 2.5 mg PO once a day (OD), insulin glargine 12 units (U) subcutaneous (SubQ) OD, and no asthma inhalers. She presented for her regular HD session, complaining of an ulcer on the lateral aspect of her right thigh which was measured as 2 × 3 cm and she was managed with topical mupirocin, analgesic gels, and dressing every other day. Two weeks later, she developed another ulcer, which was measured as 2 × 3 cm on her left thigh and was managed with the same treatment (Figure 1).

**Figure 2:**
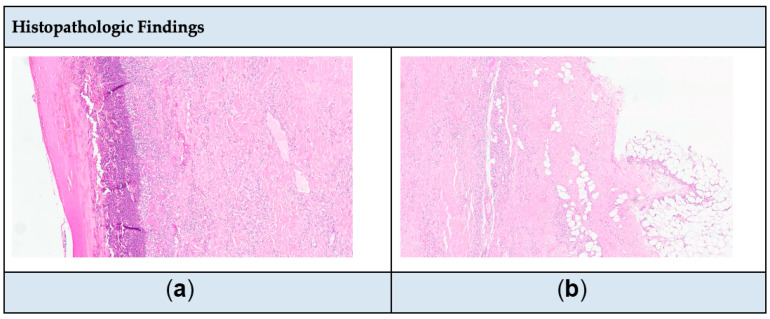
(**a**) Epidermal necrosis with dermal inflammatory infiltrate. (**b**) Inflammatory infiltrate in deep dermis and subcutis. **Note:** Microscopic examination of a tissue sample from a lesion shows full-thickness epidermal necrosis and ulceration with associated surface neutrophilic crust (**a**). The dermis contains a patchy mixed lymphohistiocytic inflammatory infiltrate with perivascular and periadnexal accentuation, with focal extension into the subcutis (**b**). No calcium deposition, fibrinoid necrosis of vessels, or well-defined granulomas are seen.

**Figure 3:**
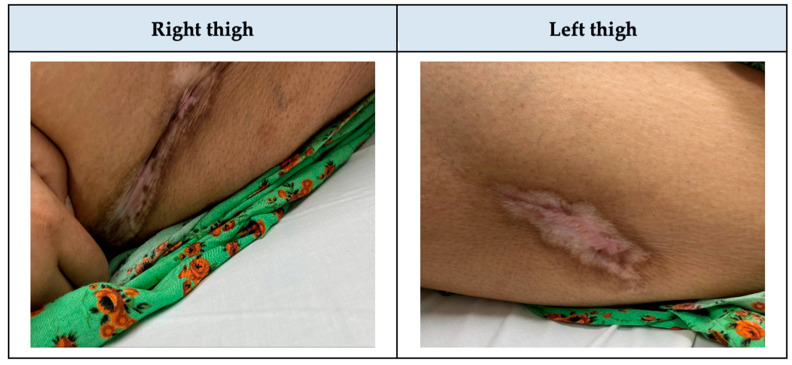
Lateral thighs after six months of calciphylaxis treatment.

When the patient presented again to her HD session, she was complaining of severe pain due to her ulcers and the decision was to admit her to the nephrology department. The dermatology team was consulted, and they ordered a skin biopsy from the right thigh to confirm the suspicion of calciphylaxis, as the patient was on hemodialysis and had more than two painful ulcers that were unresponsive to topical treatment. However, the biopsy result returned inconclusive for calciphylaxis. Then, the patient was admitted due to severe pain and for a repeated biopsy from the right thigh. Upon admission under nephrology, ulcers were observed on the lateral aspect of both thighs, accompanied by signs of collection, tenderness, and mild erythema. Laboratory findings included sodium (Na) 136 mmol/L (reference range: 136–145 mmol/L), potassium (K) 4 mmol/L (reference range: 3.5–5.1 mmol/L), calcium (Ca) 2.15 mmol/L (reference range: 2.2–2.5 mmol/L), adjusted Ca 2.33 mmol/L (reference range: 2.2–2.5 mmol/L), phosphorus (P) 0.94 mmol/L (reference range: 0.74–1.52 mmol/L), Ca × P 3.8 (reference range: <4.4 mmol/l), parathyroid hormone (PTH) 34.9 pmol/L (reference range: 2–12.5 pmol/L), chlorid (Cl) 98 mmol/L (reference range: 98–107 mmol/L), white blood cell (WBC) 10.8 × 10^9^/L (reference range: 4–11 × 10^9^/L), serum creatinine (Scr) 410 µmol/L (reference range: 44–97 umol/L), blood urea nitrogen (BUN) 7.4 mmol/L (reference range: 3.5–7.2 mmol/L), estimated glomerular filtration rate (eGFR) 11 mL/min/1.73 m^2^, high sensitivity C- reactive protein (CRP-HS) 310 mg/L (reference range: ~5 mg/L), and albumin 31 g/L (reference range: 32–46 g/L). The Wong–Baker pain scale ranged from 6 to 9. The treatment plan included discontinuing potential offending agents, such as calcium-based supplements and vitamin D. Warfarin was also discontinued since the patient had a provoked DVT and completed the anticoagulation therapy duration. Additionally, adjustments were made to the hemodialysis settings by using a low-calcium dialysate. Moreover, the patient was started on diphenhydramine 25 mg IV pushed BID for three days, amitriptyline 15 mg PO BID, and heparin 5000 U SubQ BID. Pain management strategies included acetaminophen 1 g IV OD, fentanyl IV 10 mcg OD, and pregabalin 75 mg PO BID. The team decided to perform a second biopsy and initiate IV STS therapy. The second biopsy revealed no evidence of calcium deposition or necrosis (Figure 2a,b). However, because IV STS was unavailable at the hospital, the patient was transferred to another branch of the hospital in a different city and readmitted under internal medicine to initiate treatment with IV STS. During the second admission, a CT scan of the patient’s femur/thigh showed moderate bilateral prepatellar bursitis with prepatellar and lateral deep subcutaneous fluid on the lateral aspect of the knee. The wound culture was positive for *Pseudomonas aeruginosa* and was susceptible to piperacillin/tazobactam, ciprofloxacin, and ceftazidime. Laboratory findings were as follows: Na 137 mmol/L, K 4.4 mmol/L, Ca 2.19 mmol/L, adjusted Ca 2.37 mmol/L, Cl 96 mmol/L, P 1.21 mmol/L, WBC 12.4 × 10^9^/L, Platelet (Plt) 405 × 10^3^/mcL (reference range: 150–400 × 10^3^/mcL), Scr 619 µmol/L, BUN 15.2 mmol/L, eGFR 7 mL/min/1.73 m^2^, uric acid 425 µmol/L (reference range: 210–420 umol/L), and albumin 31 g/L. The treatment plan included the administration of IV STS 25 g over 60 min administered 3/wk after HD session for 2 weeks [9], piperacillin/tazobactam 2.25 g IV Q 8 h for 7 days, amitriptyline 15 mg PO BID, and heparin 5000 U SubQ BID. The pain management regimen consisted of acetaminophen/codeine 300 mg/30 mg PO OD, hydromorphone 0.4 mg IV push every 4 hours, tramadol 50 mg IV push BID, and pregabalin 75 mg PO in the morning, and 150 mg PO at night. When the patient returned to our hospital to complete the remaining treatment course of IV STS, she was complaining of a burning sensation in her arm. Then, the infusion rate was extended over 2 h (Table 1). To facilitate further ulcer healing, the total treatment duration with IV STS was continued for 6 months (Figure 3). Notably, the patient tolerated the therapy well, with no reported adverse drug events throughout treatment.

## 3. Discussion

In this case, we presented a 42-year-old female patient on HD who developed calciphylaxis, a rare and life-threatening condition with an atypical presentation of non-healing ulcers. The absence of a clear guideline for treating calciphylaxis presents significant challenges, particularly when the presentation deviates from typical manifestations, and standard skin biopsy results are inconclusive. The proposed diagnostic criteria for calciphylaxis include the presence of all three clinical criteria or two clinical criteria plus positive histology (Table 2) [10]. Current literature suggests various management strategies, highlighting the complexity and variability in disease presentation. These strategies include wound care, hyperbaric oxygen therapy, adjustments to the dialysate bath, and discontinuation of medications such as warfarin, calcium-based supplements, vitamin D, and iron therapy. Additional interventions may involve initiating intravenous sodium thiosulfate (STS), using calcimimetics for hyperparathyroidism, and administering phosphate binders in cases of elevated phosphorus levels [8]. Unlike previous reports, where skin biopsy results frequently confirm the diagnosis, our case involved two inconclusive skin biopsies. Two studies reported patients similar to our study, with diagnoses made based on clinical criteria [11,12]. Before initiating sodium thiosulfate therapy, several treatment strategies were employed, guided by recommendations from previous case reports. These initial approaches included discontinuing warfarin, calcium-based supplements, and vitamin D, as these agents are known to contribute to vascular calcification [13,14,15,16]. Studies show that warfarin contributes to vascular calcification by inhibiting matrix Gla protein, a vitamin K-dependent protein responsible for preventing calcium buildup in the arteries [17]. Additionally, warfarin use for at least 3 years could cause calciphylaxis when combined with the other risk factors [18]. The patient’s hemodialysis regimen was adjusted to utilize a low-calcium dialysate to reduce further calcium deposition. A regimen of analgesics, including tramadol, hydromorphone, morphine, and pregabalin, were administered to manage the severe pain associated with the ulcers. Diphenhydramine, an antihistamine, was also included in the therapeutic strategy to alleviate symptoms. Despite these interventions, the patient’s ulcers persisted without significant improvement, demonstrating the refractory nature of calciphylaxis in this case and the need for alternative therapeutic options.

Despite the lack of both randomized trials and systematic reviews in hemodialysis patients, the decision to initiate sodium thiosulfate (STS) was made based on its emerging role in the literature as an effective therapy for calciphylaxis, particularly in cases where conventional treatment has failed to achieve satisfactory healing. STS is thought to work by chelating calcium deposits and promoting the solubilization of calcium salts, thereby reducing vascular calcification [10]. STS administration can cause nausea, vomiting, and skin irritation. The average treatment duration for STS was 2 to 8 weeks or until the ulcer resolved [9]. In our case, STS was administrated at a dosage of 25 g intravenously, infused over 1 hour, 3 times per week following each hemodialysis session (Table 1). However, the patient experienced a burning sensation during drug administration, so the infusion duration was extended to 2 h for a total treatment period of 6 months, leading to a marked improvement in ulcer healing and a reduction in the burning sensation. While histopathological findings (e.g., skin biopsy) are valuable in diagnosing calciphylaxis, this case highlights the importance of clinical criteria in making the diagnosis when skin biopsy results are negative. The clinical criteria for this patient was the presence of ESRD on HD, more than two painful ulcers with purpura on the buttocks and right and left thighs, with a lack of response to conventional treatments. Additionally, our observation provides a unique contribution by demonstrating that the duration of IV STS treatment can be extended safely when no clinical improvement is observed from the previous studies [10,13,14,15,16]. Our experience reinforces the safety and potential benefits of longer treatment durations in specific cases. This finding may help to guide clinicians in managing similarly complex cases where conventional treatments fail to yield rapid results.

## 4. Conclusions

This case highlights the importance of clinical judgment in the diagnosis and management of calciphylaxis, particularly when histopathological diagnostic methods yield inconclusive results. Clinical criteria, alongside a thorough assessment of the patient’s history and presentation, are vital for achieving a timely diagnosis in such challenging cases. The successful use of sodium thiosulfate in this patient adds to the growing body of evidence supporting its use as a potential treatment for calciphylaxis, especially in cases that do not respond to conventional therapy. Our findings emphasize the need for further studies to establish definitive protocols for the management of calciphylaxis.

## Figures and Tables

**Table 1:** STS Administration Timeline.

Month	Standard STS Administration Protocol [9]	Our Protocol for STS Administration	Side Effects and Tolerability
First	STS administration in dialysis patients is 25 g IV diluted in 100 mL of NaCl 0.9%, administered over 30–60 minutes, 3/wk during the last hour or after the hemodialysis session.	STS administration in our patient was **25 g IV diluted in 100 mL of NaCl 0.9%, administered over one hour,** 3/wk after the hemodialysis session.	Patient reported burning sensation in the arm during infusion.
Second to sixth		STS administration in our patient was **25 g IV diluted in 100 mL of NaCl 0.9%, extended infusion to two hours,** 3/wk after the hemodialysis session.	Patient exhibited excellent tolerance throughout the treatment course, with no adverse events reported and completed ulcers healing.

Abbreviations: STS: sodium thiosulfate; NaCl: sodium chloride.

**Table 2:** Calciphylaxis Diagnostic Criteria [10].

	Clinical Criteria	Histopathology Criteria
Standard Criteria	- ESRD on HD. - Presence of two or more painful ulcers associated with purpura, and the presence of painful ulcers located on the trunk, limbs, or penis, with lack of response to treatment.	- Necrosis and skin ulceration with calcification of the medial layer and internal elastic membrane of small and medium-sized arteries of the dermis and subcutaneous tissue.
Our Patient Criteria	- ESRD on HD. - Presence of more than two painful ulcers associated with purpura, and the presence of painful ulcers located on the buttocks, right and left thighs with a lack of response to treatment.	- Both skin biopsy revealed no evidence of calcium deposition or necrosis (calciphylaxis).

Abbreviations: ESRD: end stage renal disease; HD: hemodialysis.

## Data Availability

The data presented in this study are available on request from the corresponding author. The data are not publicly available due to privacy considerations and ethical guidelines.

## References

[1] Nigwekar S.U., Thadhani R., Brandenburg V.M. (2018). Calciphylaxis. N. Engl. J. Med..

[2] Westphal S.G., Plumb T. (2024). Calciphylaxis. StatPearls.

[3] Fine A., Zacharias J. (2002). Calciphylaxis Is Usually Non-Ulcerating: Risk Factors, Outcome and Therapy. Kidney Int..

[4] Weenig R.H., Sewell L.D., Davis M.D.P., McCarthy J.T., Pittelkow M.R. (2007). Calciphylaxis: Natural History, Risk Factor Analysis, and Outcome. J. Am. Acad. Dermatol..

[5] Yerram P., Chaudhary K. (2014). Calcific Uremic Arteriolopathy in End Stage Renal Disease: Pathophysiology and Management. Ochsner J..

[6] Nigwekar S.U., Zhao S., Wenger J., Hymes J.L., Maddux F.W., Thadhani R.I., Chan K.E. (2016). A Nationally Representative Study of Calcific Uremic Arteriolopathy Risk Factors. J. Am. Soc. Nephrol..

[7] Angelis M., Wong L.L., Myers S.A., Wong L.M. (1997). Calciphylaxis in Patients on Hemodialysis: A Prevalence Study. Surgery.

[8] Nigwekar S.U., Kroshinsky D., Nazarian R.M., Goverman J., Malhotra R., Jackson V.A., Kamdar M.M., Steele D.J.R., Thadhani R.I. (2015). Calciphylaxis: Risk Factors, Diagnosis, and Treatment. Am. J. Kidney Dis..

[9] Generali J.A., Cada D.J. (2015). Sodium Thiosulfate: Calciphylaxis. Hosp. Pharm..

[10] Jiménez-Gallo D., Ossorio-García L., Linares-Barrios M. (2015). Calcinosis Cutis and Calciphylaxis. Actas Dermo-Sifiliográficas Engl. Ed..

[11] Dardeno M., Sparling J.D., Monaco W. (2021). Warfarin-induced non-uremic calciphylaxis mimicking vasculitis: A case report. The Rheumatologist [Internet].

[12] Omer M., Bhat Z.Y., Fonte N., Imran N., Sondheimer J., Osman-Malik Y. (2021). Calcific Uremic Arteriolopathy: A Case Series and Review from an Inner-City Tertiary University Center in End-Stage Renal Disease Patients on Renal Replacement Therapy. Int. J. Nephrol..

[13] Rrapi R., Chand S., Gabel C., Ko L., Moore K.J., Steele D., Kroshinsky D. (2021). Early Diagnosis and Intervention of Calciphylaxis Leading to Rapid Resolution. JAAD Case Rep..

[14] Mihailescu M., Mehlis S. (2021). An Unusual Case of Calciphylaxis in a Psoriatic Patient without Kidney Disease. JAAD Case Rep..

[15] Gonzalez D.E., Foresto R.D., Maldonado A.L.S., Padilha W.S.C., Roberto F.B., Pereira M.E.V.d.C., Durão Junior M.d.S., Carvalho A.B. (2020). Multiple Extremity Necrosis in Fatal Calciphylaxis: Case Report. Braz. J. Nephrol..

[16] Hall D.J., Gentile L.F., Duckworth L.V., Shaw C.M., Singhal D., Spiguel L.R.P. (2016). Calciphylaxis of the Breast: A Case Report and Literature Review. Breast J..

[17] Schurgers L.J., Cranenburg E.C.M., Vermeer C. (2017). Matrix Gla-Protein: The Calcification Inhibitor in Need of Vitamin K. Thromb. Haemost..

[18] Yu W.Y.-H., Bhutani T., Kornik R., Pincus L.B., Mauro T., Rosenblum M.D., Fox L.P. (2017). Warfarin-Associated Nonuremic Calciphylaxis. JAMA Dermatol..

